# Noncoding RNA Expression Aberration Is Associated with Cancer Progression and Is a Potential Biomarker in Esophageal Squamous Cell Carcinoma

**DOI:** 10.3390/ijms161126060

**Published:** 2015-11-24

**Authors:** Hidetaka Sugihara, Takatsugu Ishimoto, Keisuke Miyake, Daisuke Izumi, Yoshifumi Baba, Naoya Yoshida, Masayuki Watanabe, Hideo Baba

**Affiliations:** 1 Department of Gastroenterological Surgery, Graduate School of Medical Science, Kumamoto University, Kumamoto 860-8556, Japan; hidetaka1125@hotmail.com (H.S.); taka1516@kumamoto-u.ac.jp (T.I.); technical.assistant.kugs01@gmail.com (K.M.); apple_morphine@yahoo.co.jp (D.I.); baba@kumamoto-u.ac.jp (Y.B.); nyoshida@kumamoto-u.ac.jp (N.Y.); 2 Cancer and Stem Cell Biology, Duke-National University of Singapore (NUS) Graduate Medical School Singapore, Singapore 169857, Singapore; 3 Department of Gastroenterological Surgery, The Cancer Institute Hospital of Japanese Foundation For Cancer Research (JFCR), Tokyo 135-8550, Japan; masayuki.watanabe@jfcr.or.jp

**Keywords:** esophageal squamous cell carcinoma, microRNA, long noncoding RNA, cancer initiation, cancer development, biomarker

## Abstract

Esophageal cancer is one of the most common cancers worldwide. Esophageal squamous cell carcinoma (ESCC) is the major histological type of esophageal cancer in Eastern Asian countries. Several types of noncoding RNAs (ncRNAs) function as key epigenetic regulators of gene expression and are implicated in various physiological processes. Unambiguous evidence indicates that dysregulation of ncRNAs is deeply implicated in carcinogenesis, cancer progression and metastases of various cancers, including ESCC. The current review summarizes recent findings on the ncRNA-mediated mechanisms underlying the characteristic behaviors of ESCC that will help support the development of biomarkers and the design of novel therapeutic strategies.

## 1. Introduction

Esophageal cancer (EC) is known as one of the most aggressive cancers, as reflected by an overall survival rate of 10%–20%. EC is the eighth most common cancer and the sixth most common cause of cancer death [1]. In EC cases diagnosed at an advanced stage, the overall five-year survival rate is 9%–40% [2]. EC is divided into two histological types, adenocarcinoma and squamous cell carcinoma. Although the incidence of esophageal adenocarcinoma is increasing, esophageal squamous cell carcinoma (ESCC) is more predominant in East Asia, including Japan. Despite improved development of multimodal techniques, such as surgery, chemotherapy and radiotherapy, the survival rate of ESCC patients remains poor because of a high incidence of local invasion and distant metastasis [3,4]. Therefore, identifying the mechanisms underlying these processes is critical for the development of biomarkers and therapeutic targets.

The human transcriptome contains numerous protein-coding messenger RNAs (mRNAs), as well as plenty of non-protein-coding transcripts. Among several kinds of noncoding RNAs (ncRNAs), long noncoding RNAs (lncRNAs) and microRNAs (miRNAs) have received attention in recent years. lncRNAs are a new class of ncRNAs that are longer than 200 nucleotides and regulate the expression levels of target genes in diverse biological processes, including chromatin modification, transcriptional and post-transcriptional levels [5,6,7]. Furthermore, recent studies have demonstrated that the expression of many lncRNAs is dysregulated, and they play critical roles in tumorigenesis and tumor progression in various types of cancer [8,9]. miRNAs are shorter noncoding RNAs (21–23 nucleotides) that bind to the 3′-untranslated region (UTR) of their target mRNAs to post-transcriptionally repress their translation. miRNAs have been involved in various pathological conditions, such as neurological diseases, cardiovascular disease, viral infection and cancer. A number of miRNAs target particular oncogenes or tumor suppressors and function in the pathogenesis of many cancers [10,11,12].

Accumulating evidence indicates that ncRNA dysregulation in ESCC plays an important role in regulating specific cellular processes, such as differentiation, proliferation, apoptosis and stress response. In addition, expression of these ncRNAs has the potential to serve as a useful biomarker for diagnosis and prognosis prediction in ESCC patients. In this review, we focus on the ncRNA-mediated mechanisms underlying tumor progression and the identification of diagnostic and prognostic prediction biomarkers in ESCC.

## 2. Dysregulation of miRNAs Involved in ESCC Development

**Figure 1:**
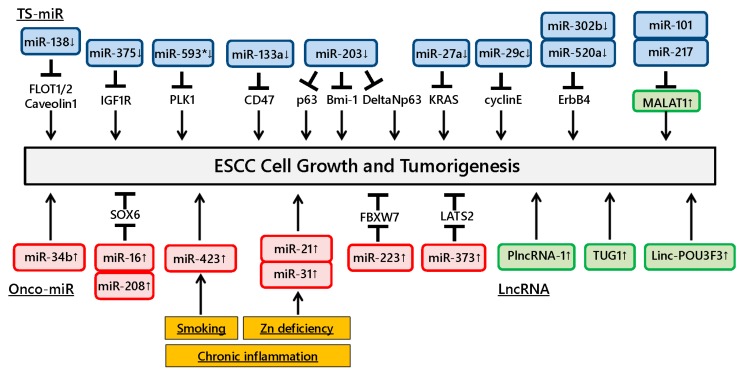
Dysregulation of ncRNAs involved in esophageal squamous cell carcinoma (ESCC) development. Dysregulation of various ncRNAs affects tumor growth in ESCC. Chronic inflammation induces ncRNA alterations in esophageal mucosa and affects downstream target gene regulation, thus contributing to ESCC development.

Dysregulation of miRNAs has been shown to have an effect on tumor growth in ESCC (Figure 1). The development of ESCC is closely related to lifestyle habits, such as tobacco smoking and/or alcohol drinking, and chronic stimuli induce genetic and epigenetic alterations in normal esophageal mucosa. Several miRNAs have been reported to be implicated in lifestyle habit-related ESCC. Wang *et al.* showed that smoke exposure increased the risk for ESCC to induce single nucleotide polymorphisms in miR-423 [13]. Zinc deficiency (ZD) is also implicated in the development of ESCC [14]. A previous study showed that miR-31 and miR-21 overexpression caused by ZD was associated with inflammation and resulted in ESCC development in a rat model [15].

Ito *et al.* showed that miR-593* directly suppressed Polo-like kinase 1 expression and reduced ESCC growth [16]. Other studies demonstrated that miR-133a and miR-375 were downregulated in ESCC tissues and significantly inhibited tumorigenesis and growth through directly targeting *CD47* and *IGF1R*, respectively [17,18]. Likewise, miR-29c was significantly downregulated in ESCC tissues and decreased tumor growth by causing cell cycle arrest mainly through suppressing cyclin E expression [19]. miR-302b and miR-520a were downregulated in ESCC and suppressed cell proliferation via binding the 3′-UTR of ErbB4 mRNA [20,21]. Furthermore, a recent study showed that miR-27a was downregulated and directly targeted the *KRAS* gene in ESCC cell lines and tissues, resulting in inhibited cell growth of ESCC [22]. Gong *et al.* found that downregulation of miR-138 induced lipid raft formation via upregulating multiple components of lipid rafts, including FLOT1, FLOT2 and caveolin-1, resulting in NF-κB activation and promotion of ESCC aggressiveness *in vitro* and *in vivo* [23].

Several studies have demonstrated the functional roles of miR-203 in ESCC progression. Yu *et al.* examined the expression of the stem renewal factor Bmi-1 and miR-203 in ESCC side population (SP) and non-SP (NSP) cells and found that Bmi-1 was increased and miR-203 was decreased in SP cells compared to NSP cells. The authors also found that the SP cell fraction and colony formation were remarkably decreased in miR-203-overexpressing cells [24]. Yuan *et al.* reported that cell growth was inhibited in ESCC cells transfected with miR-203 mimic and ΔNp63 small interfering RNA, indicating that miR-203 could suppress cell proliferation in ESCC cells through the ΔNp63-mediated signaling pathway [25]. Another study reported downregulated miR-203 expression in ESCC tissues and showed its involvement in ESCC cell growth *in vitro* and *in vivo* by regulating p63 expression [26].

In contrast, miR-34b and miR-373 were significantly overexpressed in ESCC tissues and promoted ESCC cell growth, and studies showed that miR-373 suppresses the expression of the large tumor suppressor, homolog 2 [27,28]. Likewise, other studies revealed that miR-16 and miR-208 were overexpressed in ESCC tissues and could promote cell proliferation by downregulating SOX6 [29,30]. Kurashige *et al.* demonstrated that miR-223 was upregulated in ESCC tissues and modulated the activity of F-box and WD repeat domain-containing 7, a cell cycle regulatory gene, leading to abnormal accumulation of c-Myc expression [31]. These findings suggest that the target gene regulation by various miRNAs is closely correlated with ESCC development and growth.

## 3. miRNA Regulation of Resistance to Anticancer Drugs

Understanding the mechanisms underlying drug resistance can lead to the development of novel therapeutic strategies in ESCC patients. Several miRNAs have been reported to regulate the resistance to anticancer drugs against ESCC. The expression levels of let-7b and let-7c were altered in cisplatin-resistant ESCC cells, and let-7c directly repressed the cisplatin-activated interleukin (IL)-6/STAT3 pro-survival pathway, leading to poor prognosis in ESCC patients [32]. Overexpression of miR-218 resulted in suppressed cell growth, colony formation, migration and invasion, caused cell apoptosis and arrested cell cycle in the G0/G1 phase. miR-218 mimics increased the sensitivity to the anti-tumor effect of cisplatin in ESCC cell lines through regulating the expression of phosphorylated PI3K, AKT and mTOR [33]. These results indicate that these miRNAs act as tumor-suppressive (TR) miRs related to the drug resistance of ESCC.

Conversely, previous studies have demonstrated the involvement of oncogenic miRNAs (onco-miRs) in the drug resistance of ESCC. One report showed that miR-141 induced resistance to cisplatin-induced apoptosis through targeting YAP1, and another study showed that miR-200c repressed PPP2R1B, a subunit of protein phosphatase 2A, and was also involved in drug resistance through the Akt pathway in ESCC cells [34,35]. Downregulation of miR-27a and miR-296 conferred sensitivity of both *P*-glycoprotein-related and *P*-glycoprotein-nonrelated drugs and promoted adriamycin-induced apoptosis by suppressing MDR1 expression [36,37]. These previous findings may show possible candidates for the development of individualized treatment against refractory ESCC.

## 4. miRNAs Involved in ESCC Cell Migration and Invasion

The motility of cancer cells is essential for invasion into blood vessels and spreading to the surrounding organs. One of the most common processes that induces the early steps of cancer metastasis is epithelial to mesenchymal transition (EMT). The expressions of miR-9, miR-25 and miR-92a, which regulate CDH1 expression, were shown to be upregulated in ESCC tissues and to promote cell migration and invasion [38,39,40]. In addition, Zhang *et al.* reported a positive association between miR-21 expression and cigarette smoking. Upregulation of miR-21 was also induced by nicotine in an ESCC cell line, promoting EMT via transforming growth factor-β [41]. However, miR-205 and the miR-200 family suppressed tumor activities by EMT inhibition through targeting ZEB expression in ESCC cells [42,43].

Several studies have identified miRNAs that promote ESCC cell invasion. miR-21 and miR-183 promote ESCC cell growth and invasion through targeting PDCD4 [44,45]. Ohta *et al.* reported that the expression of miR-328, a candidate regulator of GNG7 mRNA, was inversely and significantly associated with GNG7 expression in 16 ESCC cell lines, suggesting that miR-328 could repress GNG7, leading to the invasiveness of ESCC cells and poor prognosis [46]. Li *et al.* showed that miR-21 was overexpressed in ESCC tissues and cell lines. Furthermore, knockdown of miR-21 significantly increased the expression of PTEN protein and consequently reduced cell proliferation, invasion and migration [47]. Tian *et al.* revealed that overexpression of miR-10b in ESCC cells increased cell motility and invasiveness induced by the suppression of endogenous KLF4 protein [48].

In contrast, other studies have identified miRNAs that suppress ESCC cell invasion. The expression of miR-100 was downregulated in ESCC tissues, and miR-100 modulated cell invasion through the repression of mTOR expression [49]. Overexpression of miR-625 inhibits cell proliferation and invasion through the repression of Sox2 [50]. miR-326 is involved in the regulation of VEGF-C-mediated cortactin expression and the subsequent invasion of ESCC cells [51]. Knockdown of FSCN1 or matrix metalloproteinase 14, one of the invadopodia-related proteins, and overexpression of miR-133a inhibited the growth and invasion of ESCC cells [52]. Ectopic expression of miR-195 induced G1 cell cycle arrest, leading to a significant decrease in cell growth, migration and invasion through the suppression of Cdc42 [53]. Zhang *et al.* showed that the overexpression of miR-200b or knockdown of Kindlin-2 in ESCC cells suppressed cell protrusion and focal adhesion (FA) formation and decreased cell spreading and invasiveness/migration. Furthermore, the authors demonstrated that Rho-family guanosine triphosphatases and FA kinase had an impact on the biological effects of the miR-200b-Kindlin-2 cascade [54].

**Figure 2:**
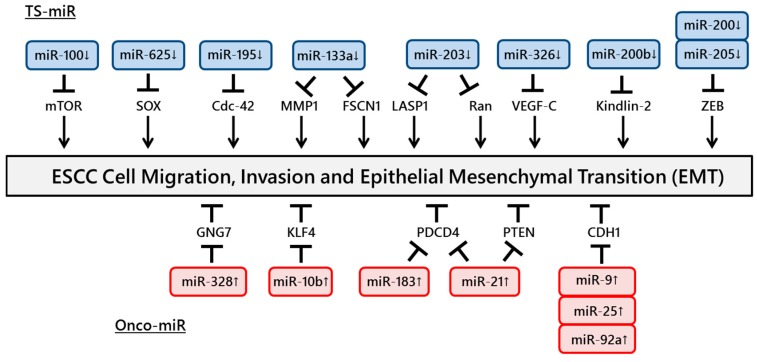
ncRNAs involved in ESCC cell migration and invasion. A number of ncRNAs regulate ESCC cell migration and invasion through targeting tumor suppressor genes or oncogenes.

Several studies have demonstrated important functions of miR-203. Takeshita *et al.* reported downregulation of miR-203 expression in ESCC tissues and showed that miR-203 played an important role in ESCC cell invasion by regulating LIM and SH3 protein 1 expression [55]. Another study revealed that overexpression of miR-203 in ESCC cells remarkably induced cell apoptosis and inhibited cell growth, migration and invasion via targeting small GTPase Ran [56]. The miRNAs involved in ESCC cell migration and invasion are summarized in Figure 2.

## 5. miRNAs as Diagnostic and Prognostic Biomarkers in Primary ESCC Tissue

The identification of effective diagnostic and prognostic prediction biomarkers is invaluable for the development of treatment strategies against ESCC (Table 1). Among the members of the miR-17–92 cluster, which have been reported to be highly expressed in several cancers, miR-17, miR-18a and miR-19a serve as potential unfavorable prognostic biomarkers and are associated with some clinicopathologic factors [57]. miRNA microarray analysis using 31 ESCC samples showed that high expressions of miR-103 and miR-107 were associated with poor prognosis in overall survival by multivariate analysis [58]. Fu *et al.* investigated 22 studies including a total of 1946 participants by searching PubMed, Embase and Web of Science to identify miRNAs that may function as prognostic biomarkers in ESCC. Among 33 prognostic miRNAs, miR-21 and miR-375 were selected, and further analysis revealed that upregulation of miR-21 and downregulation of miR-375 can predict unfavorable prognosis in ESCC [59].

## 6. Plasma miRNAs as Noninvasive Biomarkers

The identification of novel biomarkers in plasma/serum miRNA is required for early detection and prognostic prediction in ESCC (Table 1). A previous study showed that plasma concentrations of miR-19b and miR-25 were significantly higher in ESCC patients than in healthy volunteers, and plasma miR-25 levels were significantly downregulated in postoperative samples compared to preoperative samples and were significantly overexpressed in association with ESCC recurrence [60]. Another report showed that serum miR-1246 was markedly upregulated in ESCC patients and has the possibility of being a novel noninvasive biomarker for the early detection of ESCC [61]. Plasma concentrations of miR-18a, miR-21 and miR-375 were significantly higher in ESCC patients compared to healthy volunteers, and plasma levels of miR-18a were significantly lower in postoperative samples compared to preoperative samples [62,63]. Furthermore, the serum levels of miR-200c in ESCC patients were significantly higher than those in healthy volunteers, and high expression of miR-200c was significantly associated with poor response to chemotherapy [64].

In addition to plasma miRNAs, recent studies have focused on the detection of exosomal miRNAs. Tanaka *et al.* reported that the levels of miR-21 in exosomes were higher in patients with ESCC than those in the control group, and exosomal miR-21 expression was associated with advanced tumor stage, positive lymph node status and the presence of metastasis [65]. Taken together, this evidence shows that the search for definitive noninvasive biomarkers in plasma/serum is still in the preliminary stages, and further prospective studies are required in the future.

## 7. Long Noncoding RNA Aberration in ESCC

Several lncRNAs have been reported to function as potential oncogenes in ESCC (Figure 1). Metastasis associated with lung adenocarcinoma transcript 1 (MALAT1), a novel lncRNA, showed a significant impact on proliferation, invasion and metastasis of ESCC cells. Tumor-suppressive miRNAs (TS-miRs), miR-101 and miR-217, could suppress MALAT1 expression through posttranscriptional regulation [66]. The expression of PlncRNA-1 and taurine upregulated gene 1 (*TUG1*) were significantly upregulated in human ESCC compared to the adjacent normal tissues and induced ESCC cell proliferation [67,68]. Furthermore, long intergenic non-protein coding RNAs (lincRNAs) have been explored, and linc-POU3F3 was significantly upregulated in ESCC compared to corresponding normal tissues. RNA immunoprecipitation assays showed that linc-POU3F3 was associated with the enhancer of zeste homolog 2 (EZH2) mRNA. Overexpression of linc-POU3F3 in ESCC cell lines increased their proliferation, colony formation ability and tumorigenicity [69].

Several lncRNAs have the potential of being noninvasive biomarkers in plasma/serum for early detection and prognostic prediction of cancer (Table 1). Previous studies have shown that lncRNA HOX transcript antisense RNA (HOTAIR) was upregulated in ESCC tissues compared to the controls and correlated with the poor prognosis of ESCC patients. In addition, the functional investigation showed that HOTAIR contributed to the malignant behavior of ESCC cells, such as proliferation, anti-apoptosis, migration and invasion, through regulating diverse gene expression [70,71,72,73]. A recent study further identified a novel intronic HOTAIR enhancer and a functional ESCC susceptibility SNP rs920778 in Chinese populations [74]. Furthermore, several studies demonstrated that SPRY4-IT1, CCAT2 and prostate cancer-associated ncRNA transcript 1 (PCAT-1) were upregulated in ESCC tissues compared to the adjacent noncancerous tissues, and high expression of these lncRNAs was significantly associated with the clinical pathological stage and poor survival rate of ESCC [75,76,77]. On the other hand, LOC285194 expression was significantly downregulated in ESCC tissues and cell lines, and low expression of LOC285194 had a relationship with chemoradiotherapy resistance and unfavorable prognosis [78].

Plasma lncRNAs have also been explored, and the levels of POU3F3, HNF1A-AS1 and SPRY4-IT1 were significantly upregulated in plasma from ESCC patients. Among the three lncRNAs, POU3F3 showed the most reliable potential for detecting ESCC, suggesting that plasma POU3F3 could be a novel biomarker for the diagnosis of ESCC [79].

**Table 1:** Potential biomarkers in ESCC.

Materials	Non-coding RNA Signature	Potential Value	Reference
Primary tissues	miR-17 ↑, miR-18a ↑, miR-19a ↑	Prognostic factor	[57]
	miR-103 ↑, miR-107 ↑	Prognostic factor	[58]
	miR-21 ↑, miR-375 ↑	Prognostic factor	[59]
	HOTAIR ↑	Prognostic factor	[70,71,72,73,74]
	SPRY4-IT1 ↑	Prognostic factor	[75]
	CCAT2 ↑	Prognostic factor	[76]
	PCAT-1 ↑	Prognostic factor	[77]
	LOC285194 ↓	Prognostic factor; responsiveness to chemoradiotherapy	[78]
Plasma	miR-19b ↑, miR-25 ↑	Screening marker	[60]
	miR-1246 ↑	Screening marker	[61]
	miR-18a ↑	Screening marker	[62]
	miR-21 ↑, miR-375 ↑	Screening marker	[63]
	miR-200c ↑	Screening marker; responsiveness to chemoradiotherapy	[64]
	POU3F3 ↑	Screening marker	[79]
Exosome	miR-21 ↑	Correlation with tumor progression	[65]

## 8. Conclusions

Accumulating evidence has suggested that ncRNAs play important roles in the initiation and development of ESCC. ESCC development is likely to be influenced by lifestyle factors, such as tobacco smoking and alcohol drinking, and these factors cause not only genomic alterations, but also dysregulation of various ncRNAs. Furthermore, the complex dysregulation of various ncRNAs is involved in the metastatic processes of ESCC cells. Previous studies for ncRNAs have reported a one-to-one relationship with a certain target gene, but actually, the ncRNA interacts with various genes. By establishing the model *in vivo* that genetically regulates the ncRNA expression, it will be possible to identify the most important ncRNA having an effect on ESCC development. This will lead to the design of novel strategies for ESCC treatment by targeting the ncRNA. To develop treatments that regulate distinct ncRNA-gene pathways, delivery systems for ncRNA modulators in ESCC tissue will be required before progressing to clinical application. Notably, several potential biomarkers related to ncRNAs in ESCC tissue or plasma have already been reported so far. However, clinical application of these candidates has not been accomplished at this time, because of the limit of sensitivity. Thus, further studies conducted in terms of ncRNAs are crucial to help further the development of useful biomarkers and successful therapeutic strategies against ESCC.
